# Postoperative pain pathophysiology and treatment strategies after CRS + HIPEC for peritoneal cancer

**DOI:** 10.1186/s12957-020-01842-7

**Published:** 2020-03-31

**Authors:** Xiao Wang, Tianzuo Li

**Affiliations:** Department of Anesthesiology, Beijing Shijitan Hospital, Capital Medical University, No. 10 Tieyi Road, Yangfangdian, Haidian District, Beijing, 100038 China

**Keywords:** Peritoneal cancer, Pain, Nociceptive, Neuropathic, Cytoreductive surgery, Hyperthermic intraperitoneal chemotherapy, Analgesics

## Abstract

**Background:**

Cytoreductive surgery (CRS) combined with hyperthermic intraperitoneal chemotherapy (HIPEC) is a treatment choice for peritoneal cancer. However, patients commonly suffer from severe postoperative pain. The pathophysiology of postoperative pain is considered to be from both nociceptive and neuropathic origins.

**Main body:**

The recent advances on the etiology of postoperative pain after CRS + HIPEC treatment were described, and the treatment strategy and outcomes were summarized.

**Conclusion:**

Conventional analgesics could provide short-term symptomatic relief. Thoracic epidural analgesia combined with opioids administration could be an effective treatment choice. In addition, a transversus abdominis plane block could also be an alternative option, although further studies should be performed.

## Background

Primary peritoneal cancer is a rare cancer that originates from the lining of the peritoneal cavity. Most peritoneal cancers are secondary to the dissemination of malignant cells from gastrointestinal or gynecological cancers [1]. Instead of being the terminal stage of cancer metastasis, secondary peritoneal cancer has been considered as a locoregional extension from the primary cancer [2]. The mainstay treatment for secondary peritoneal cancer is cytoreductive surgery (CRS) combined with hyperthermic intraperitoneal chemotherapy (HIPEC) [3–6]. Studies have revealed the improved survival rates of patients who received CRS + HIPEC treatment [7–9]. However, CRS + HIPEC treatment is a complex surgical procedure that commonly requires a long operation duration and causes significant surgical injuries. In addition, repeated lavages in the peritoneal cavity with high-dose thermo-chemotherapeutic agents could exaggerate the stimulations and inflammations to the peritoneum. All these could contribute to the development of severe postoperative pain after surgery. Poorly managed postoperative pain could result in elevated stress and anxiety and further affect the quality of life of patients [10]. Due to the huge injury, patients with CRS and HIPEC have a high requirement for analgesia.

Our understanding on the development and treatment of postoperative pain after CRS + HIPEC treatment continues to evolve. The present study describes the recent advances on the etiology of postoperative pain after CRS + HIPEC treatment and summarizes the treatment strategy and outcomes.

## Main text

### Pathophysiology of postoperative pain

Acute postoperative pain after CRS + HIPEC treatment is different from the pain that occurs during a traditional abdominal surgery. CRS + HIPEC treatment not only causes nociceptive pain through surgical injuries and inflammation, but also induces neuropathic pain through simulations from the thermal chemotherapy (summarized in Table 1). Many factors can influence postoperative pain perception. These factors include preoperative baseline pain intensity; intraoperative injury from surgical incisions to the skin, muscle, nerves, and bones; postoperative inflammation; and abnormal ectopic neural activities from nerve damage. Mechanical injuries during the surgery and chemical and thermal injuries from the thermo-chemotherapy could cause nociceptive pain. Local inflammation responses at the site of injury could reduce the threshold of local nerve sensitivity, resulting in inflammatory pain [20]. Nerve injury could cause neuropathic pain [21]. All of these can interact with each other and promote peripheral and central pain sensitizations [22, 23].

**Table 1:** Pathophysiology of postoperative pain

*Nociceptive pain*		
Inflammatory nociceptive pain [11, 12]	Peripheral sensitization [13]	Prostaglandin E_2_, cytokines, nerve growth factor, and substance P. DAMPs, TNF-α, IL-6, IL-8, IL-10.
	Central sensitization [14]	Microglia and inflammatory factors
*Neuropathic pain*		
Chemotherapeutic agents	Mitochondrial dysfunction and oxidative stress [15]	
	Increased calcium level	
	Activation of glutamate receptor	
	Activation of TRPV1 and TRPV4 [16]	
	Increased expression of voltage-gated sodium channels [17]	
	Aberrant expression of voltage-gated potassium channels [18]	
	Neuroinflammation	
*Chronic pain*	Nerve injury, excessive inflammatory response, abnormal immune regulation [19]	

### Nociceptive pain

#### Inflammatory nociceptive pain

Intense inflammatory responses have been reported during surgical operations. Both surgical injuries and subsequent infections could cause inflammatory nociceptive pain after CRS + HIPEC treatment. This is especially significant in patients with complications [11, 12]. High levels of serum danger-associated molecular patterns (DAMPs), tumor necrosis factor-α (TNF-α), interleukin-6 (IL-6), interleukin-8 (IL-8), and interleukin-10 (IL-10) have been identified in patients after CRS + HIPEC treatment. DAMPs could induce the local accumulation and activation of macrophages, which releases interleukin-1β (IL-1β), TNF-α, and other pro-inflammatory cytokines. All these cytokines could affect peripheral and central pain sensitization [11, 12].

#### Peripheral sensitization

Peripheral pain sensitization has been reported during the postoperative stage [13]. Prostaglandin E2, cytokines, nerve growth factor, and substance P in the surgical incision site and serum can activate and sensitize peripheral pain receptors [24]. DAMPs and other pro-inflammatory cytokines can directly or indirectly act on the receptors of nociceptive neurons and activate a variety of complex signaling pathways, including protein kinase A, protein kinase C, and p38 mitogen-activated protein kinase (MAPK). This could further reduce the peripheral neuronal excitation threshold and result in short-term peripheral sensitivity [25, 26].

#### Central sensitization

Central neuronal sensitization has been reported to be involved in postoperative hyperalgesia [14]. Pro-inflammatory cytokines, such as IL-1β, IL-6, and TNF-α, were maintained at low levels under normal situations. When surgical injury causes nerve damages, the microglia in the spinal cord and brainstem are activated by surface P2 receptors, chemokine receptors, and toll-like receptors (TLRs). The activated small microglia can release a series of inflammatory factors (IL-1β, IL-6, and TNF-α) that mediate neuroinflammatory responses, leading to central sensitization [27].

### Neuropathic pain

Trauma, infection, cancer, and other conditions could cause neuropathic pain. This can be characterized as spontaneous pain, allodynia (pain response from non-noxious stimulations), and hyperalgesia (excessive reactions from noxious stimulation) [28, 29]. Common histopathological and neurophysiological changes include neurodegeneration, loss of myelinated fibers, and the demyelination of myelinated fibers [30].

Morales-Soriano et al. analyzed the CRS + HIPEC procedure in 25 treatment centers for peritoneal cancer in Spain. The commonly used chemotherapeutic agents were platinum (cisplatin, oxaliplatin), taxanes (paclitaxel, docetaxel), and mitomycin [31]. Either the structure or the function of neurons and glial cells is changed by platinum-based chemotherapeutics [32]. There are various changes in the intracellular organelles (particularly mitochondria), membrane receptors, and ion channels, which are followed by changes in intracellular homeostasis, signaling, and neurotransmission. These alternations can lead to neuroinflammation, DNA damage, and axonal degeneration. Peripheral neuropathy is initiated by the accumulation of platinum-DNA adducts in dorsal root ganglion and trigeminal ganglion neurons, and this is probably the primary mechanism of neurotoxicity induced by platinum-based chemotherapeutics [33]. Taxanes cause the disruption of microtubules, which impairs axonal transport; leads to Wallerian degeneration; changes the activity of Na^+^, K^+^, and TRP ion channels; and induces the hyperexcitability of peripheral neurons. The mitochondrial damage promotes the production of reactive oxygen species, which damages the function of enzymes, and the structure of proteins and lipids. The disturbance of calcium homeostasis within neuronal cells causes the apoptosis and demyelination of peripheral nerves, which changes the excitability of peripheral neurons. The activation of glial cells induced by taxanes causes the activation of immune cells and the release and elevation of pro-inflammatory cytokines (interleukins and chemokines), which leads to nociceptor sensitization and the development of neuroinflammation [33–35].

Chemotherapeutic agents act on mitochondria, ion channels, and nerve structures and cause inflammation and severe NPP. Pain could persist during the entire treatment process of the chemotherapy and even last after the discontinuation of therapeutic agents [36].

### Mitochondrial dysfunction and oxidative stress

Chemotherapeutic agents cause mitochondrial damage mainly through the destruction of the ATPase-dependent sodium/potassium pump and disruptions in calcium balance. Paclitaxel can open the voltage-gated anion channels located in the outer membrane of mitochondria, leading to mitochondrial swelling and vacuolation. Oxaliplatin and other platinum-based agents can cause the attachment of platinum to mitochondrial DNA, resulting in decreased essential protein synthesis in mitochondria [37]. Mitochondrial dysfunction can induce oxidative stress, which plays an important role in the development of neuropathic pain [15]. Oxidative stress responses can release a large amount of oxidative products (oxygen-free radicals, syperoxynitroso, nitric oxide, etc.) to over-activate poly ADP-ribose polymerase (PARP). This further interferes with mitochondrial energy metabolism and exaggerates nerve damage. The expression of MAPK, NF-κB, and activator protein 1 was elevated, which induces the synthesis of pro-inflammatory factors. All these participate in the development of peripheral sensitization [32].

### Calcium channel

Chemotherapeutic agents can increase the expression of the DRG, calcium channel α2-δ1 subunit in the spinal cord dorsal horn neurons, sodium channel, and NMDA receptors. The activation of these receptors can lead to the influx of extracellular calcium and exudation of mitochondrial calcium. The increase in level of intracellular calcium can cause death in neurons through the production of oxygen free radicals and apoptosis. Calcium channels include voltage-gated calcium channels (VGCC), chemical channels (glutamate), and the transient receptor potential (TRP) family.

### Voltage-gated calcium channel

Oxalate, a metabolite of oxaliplatin, can chelate with intracellular calcium to destroy the voltage-gated ion channel [38]. This could damage the peripheral nerves, but can be inhibited by a calcium channel antagonist [39].

### Glutamate

Glutamate is an important excitatory neurotransmitter in the central nervous system. This can produce excitatory postsynaptic potentials by activating post-synaptic ionotropic glutamate receptors (NMDA receptors) in spinal dorsal horn neurons. The pain signal is transmitted to the advanced nerve center. The inflammatory reaction after tissue injury leads to the persistent activation of NMDA. The opening of the NMDA channel increases the influx of calcium, leading to central sensitization. Carozzi et al. reported the neuroprotective effect of oral glutamate carboxypeptidase inhibitors on peripheral neuropathy induced by three accepted animal models of chemotherapy (cisplatin, paclitaxel, and bortezomib) [40].

### TRP family

TRP consists of seven subfamilies with 28 channels. Among these, TRPA1, TRPV1, and TRPV4 channels are mainly expressed in the DRG and trigeminal ganglion ganglia and are associated with NPP. TRPV1 is activated by high temperature (≥ 43 °C) and oxidative stress in mammalian cells, resulting in oxidative damage and hyperalgesia [41, 42]. When the temperature becomes higher than 24 °C, TRPV4 can also be activated [43]. The temperature used for HIPEC was 43 °C, which is the critical temperature for the irreversible damage of tumor cells [44]. During this process, the activation of TRPV1 and TRPV4 could occur, resulting in postoperative NPP. In the process of removing bacteria and viruses, ROS are produced by anti-inflammatory cells, such as macrophages and microglia [16]. There is a direct relationship between increased ROS levels and inflammatory pain. ROS could also directly activate TRPV1 to cause hyperalgesia [45]. Cisplatin and oxaliplatin induce TRAP1 activation by increasing the ROS level [46]. TRPV1 and TRPA1 have a synergistic effect to activate the DRG channel [47]. Paclitaxel could activate TRPV4 to induce the elevated levels of oxidative stress and mechanical pain. The intrathecal injection of TRPV4 can relieve the paclitaxel-induced mechanical hyperalgesia [48, 49].

### Sodium channel

Studies have reported the increased expression of voltage-gated sodium channels (VGSCs) in ovarian and gastrointestinal cancers [50, 51]. These cancers are the frequent causes of secondary peritoneal cancer. VGSCs play an important role in the production and conduction of action potentials at the terminal end of pain receptors and axons. Nine subtypes of VGSCs, NaV1.1–1.9, have been identified. Among these, subtypes NaV1.7, 1.8, and 1.9 have been considered to have a close relationship with the excitability of pain nociceptors [17]. Oxaliplatin could alter the functional properties of VGSCs, resulting in extended opening duration and the hyperexcitability of sensory neurons. Paclitaxel could increase the expression of VGSC subtype NAV1.7 in the DRG. The VGSC antagonist tetrodotoxin can alleviate the neuropathic pain caused by paclitaxel [33, 52].

### Potassium channel

Voltage-gated potassium channels (VGKCs) include 12 subtypes and have aberrant expression levels in cancer tissues [18]. VGKCs are important regulators of neuronal excitability and play an important role in maintaining the membrane potentials [53]. These could control the action potential generation and regulate the release of neurotransmitters [54]. A previous study revealed that the decreased expression and activity of VGKCs is one of the causes of the peripheral sensitization of afferent nociceptive fibers and one of the major factors of persistent pain [55].

### Neuroinflammation

Chemotherapy can cause significant pathological changes in the DRG, as well as in the surrounding peripheral neurons and satellite cells. After nerve injury, immune cells (mast cells, neutrophils, macrophages, Schwann cells, and T cells) are activated and release large amounts of inflammatory factors, including pro-inflammatory factors (IL-1β, IL-6, and TNF-α), chemokines (chemokine ligand 2, CCL2), inflammatory mediators (prostaglandin E2, PGE2), histamine, bradykinin, and nerve growth factors (NGFs) [56]. The secreted inflammatory mediators increase the expression of sodium and calcium channels, causing peripheral pain sensitization. Inflammatory factors IL-1β and TNF-α can directly stimulate A-fibers and C-fibers to sensitize these and promote abnormal pain and hyperalgesia after nerve injury. Paclitaxel binds to and activates toll-like receptor 4 (TLR4) in monocytes. After 7 days of paclitaxel treatment, TLR4 became elevated in the DRG and became synchronized with the development of mechanical hypersusceptibility induced by the chemotherapy [57].

### Chronic pain

The pain intensity peaked at 3 months after the CRS + HIPEC treatment [58], and this returned to baseline levels after 9 months in 80% of patients. However, the mechanism for the chronic pain remains unclear. Some studies suggest that nerve injury caused by surgery is a necessary premise for the occurrence of postoperative chronic pain, while the excessive inflammatory response of the nervous system and abnormal immune regulation plays a key role in the progression of postoperative acute pain to chronic pain [19]. At present, no study has investigated the type and mechanism of chronic pain after CRS + HIPEC treatment.

### Pain treatment

In general, the treatment for postoperative pain after CRS + HIPEC treatment includes analgesics and regional nerve blocks (summarized in Table 2). As mentioned above, surgical injuries and chemotherapy agents could cause central and peripheral inflammation after CRS + HIPEC treatment, which could lead to both central and peripheral sensitization, and induce pain [11, 12]. The elevated baseline level of inflammation was also associated with poor prognosis [73]. In a recent meta-analysis, the perioperative use of dexmedetomidine significantly reduced the serum concentrations of IL-6, IL-8, and TNF-α [63]. For surgical patients, a single injection of an induction dose of ketamine was found to reduce the IL-6 level for 7 days [64, 65]. The use of propofol during major surgery could reduce the levels of IL-6 and IL-8 [62]. Celecoxib also reduced the prostaglandin E2 level within 48 h after endoscopic surgery [59]. However, Pascal et al. retrospectively analyzed patients with CRS + HIPEC treatment and found that there was no alleviation of perioperative inflammation with the preoperative administration of celecoxib, tramadol, and pregabalin, and intraoperative TIVA combined with propofol, dexmedetomidine, ketamine, and lidocaine [61].

**Table 2:** Treatment of postoperative pain

*Analgesic*	
Acetaminophen, NSAIDs, COX-2 inhibitor [59], gabapentin [60]	There was no alleviation of perioperative inflammation with the preoperative administration of celecoxib, tramadol and pregabalin, and intraoperative TIVA combined with propofol, dexmedetomidine, ketamine, and lidocaine [61].
Propofol [62], dexmedetomidine [63], ketamine [64, 65], and lidocaine [66] have independent anti-inflammatory properties.	
Calcium channel α-2-δ ligand anticonvulsant drugs, tricyclic antidepressants, selective 5-HT, and norepinephrine reuptake inhibitors	They provide symptomatic reliefs and the effects are often limited.
*Regional nerve block*	
TEA combined with opioids has the advantages of analgesia, early extubation after surgery, lower postoperative pulmonary complications, and reduced incidence of postoperative complications [67–70]	TEA is a safe option for CRS + HIPEC treatment, regardless of some fluctuations in intraoperative coagulation measurements [71, 72].

Conventional analgesic agents (non-steroidal anti-inflammatory drugs and opioids) have had little effect in the treatment of NPP. Medications, including calcium channel α-2-δ ligand anticonvulsant drugs, tricyclic antidepressants, selective 5-HT and norepinephrine reuptake inhibitors, and local anesthetic drug lidocaine, mainly provide symptomatic relief. These effects are often limited and can have serious side effects.

Although intravenous opioid + thoracic epidural anesthesia (TEA) is the leading choice with many benefits for pain treatment after HIPEC + CRS, the dose of opioids was higher and the duration was longer, when compared to what this was supposed to be in children with HIPEC + CRS [74]. However, this was different from the finding that the duration of opioids was shorter in adults with epidural analgesia [75]. Due to the adverse reactions of opioids (including nausea and constipation), perioperative intravenous lidocaine was used to reduce postoperative pain. However, the systematic analysis conducted by Weibel et al. revealed that it remains uncertain whether perioperative intravenous lidocaine could benefit in decreasing the early postoperative pain score, improving the gastrointestinal recovery, and reducing the postoperative nausea and the consumption of opioids, when compared with placebo or non-treatment [76].

TEA is the “gold standard” for postoperative analgesia in major abdominal surgery [60]. This has also been used for patients who received hyperthermic intrathoracic chemotherapy [77, 78]. Evidence has shown that TEA combined with opioids has the advantages of analgesia, early extubation after surgery, lower postoperative pulmonary complications, and reduced incidence of postoperative complications after CRS + HIPEC treatment [67–70]. Some studies have recommended the use of TEA to reduce the incidence of postoperative intestinal obstruction [79, 80]. Regional anesthesia also improves tumor recurrence and postoperative survival rates [81–85]. This might also reduce the incidence of chronic pain and improve patient satisfaction [86].

However, there are controversies in the implementation of TEA, since HIPCE could affect blood coagulation and cause thrombocytopenia [68]. A recent prospective clinical study indicated that TEA is a safe option for CRS + HIPEC treatment, regardless of some fluctuations in intraoperative coagulation measurements [71]. Chua et al. investigated 4277 patients who received the CRS + HIPEC treatment and found no postoperative epidural hematoma in these patients [72]. Due to the difficulty of inserting a catheter, or since traumatic surgery is the main cause of spinal cord hematoma, the preoperative assessment of a patient’s history of previous bleeding and medication history is essential and should be performed by an experienced anesthesiologist [68]. A study compared the postoperative analgesic effects of the transversus abdominis plane (TAP) block and TEA in open and laparoscopic colorectal surgery and revealed that TAP infusion was non-inferior [87].

Many factors influence postoperative pain after CRS + HIPEC treatment. The changes in these factors, such as inflammation, oxidative stress, and ion channel, can cause difficulties in providing postoperative analgesia and controlling chronic pain. At present, the management of postoperative acute and chronic pain is mainly from TEA combined with opioids. However, TEA remains challenging and is subject to many factors, such as blood coagulation status. In recent years, the TAP block has gradually been accepted by clinicians [60, 88]. This blocks the lower thoracic nerves (T7–T12) and the anterior branch of the first lumbar nerve (L1), thereby producing analgesic effects on its branches to the anterior abdominal wall skin, muscle, and parietal peritoneum. The procedure is simple with few complications. Furthermore, this can effectively reduce abdominal incision pain and decrease the requirements for opioids [89]. TAP technology can be considered for the analgesic strategy after CRS + HIPEC treatment.

Some studies have compared the postoperative analgesic effect of TAP with other analgesic methods. Lapmahapaisan et al. compared the effects of the local infiltration of 0.25% bupivacaine in a wound and TAP block by 0.25% bupivacaine. It was found that for pediatric patients undergoing massive non-laparoscopic abdominal surgery, a surgically administered TAP (sTAP) block has no distinct advantage over local infiltration and does not induce any effect in postoperative pain control [90]. Deng et al. compared the analgesic effects of the quadratus lumborum block and TAP block after laparoscopic colorectal cancer surgery. The results revealed that the quadratus lumborum block was a more effective method for postoperative analgesia and that this reduced the consumption of sufentanil. However, these literatures were all about major laparoscopic procedures, and the pain was still not comparable to HIPEC + CRS. Considering the advantages of TAP, this is still one of the best choices for CRS + HIPEC surgery [91].

## Conclusion

In summary, both nociceptive and neuropathic processes participate into the development of postoperative pain after CRS + HIPEC treatment in peritoneal cancer. Dysregulations of cytokines, nerve cell signaling pathways, and ion channels occur with these peripheral and central pain sensitizations. Conventional analgesics might offer symptomatic relief. Thoracic epidural analgesia and TAP blocks could provide better pain control.

### Future perspective

Since more and more patients are undergoing CRS + HIPEC treatment, it has become a huge challenge for anesthesiologists to determine how to provide effective postoperative analgesia and long-term pain control. Thoracic epidural analgesia has to consider the patient’s coagulation function and the anesthesiologist’s personal experience and skills, which limits its clinical applications. With the prevalence of ultrasound and the improvement of the skills of nerve blocks in anesthesiologists, the TAP block, which is easier and less traumatic, would replace thoracic epidural analgesia and become a routine postoperative analgesia method [92, 93]. At present, few studies have evaluated the long-term analgesic outcomes of the TAP block. The investigators consider that more research to study the efficacy of TAP blocks on chronic pain are warranted in the future.

## Data Availability

The datasets used and/or analyzed during the study are available from the corresponding author on reasonable request.

## Abbreviations

CRS: Cytoreductive surgery
HIPEC: Hyperthermic intraperitoneal chemotherapy
DAMPs: Danger-associated molecular patterns
TNF-α: Tumor necrosis factor-α
IL-6: Interleukin-6
IL-8: Interleukin-8
IL-10: Interleukin-10
IL-1β: Interleukin-1β
MAPK: Mitogen-activated protein kinase
TLRs: Toll-like receptors
PARP: Poly ADP-ribose polymerase
DRG: Dorsal root ganglion
VGCC: Voltage-gated calcium channels
TRP: Transient receptor potential
ROS: Reactive oxygen species
VGSCs: Voltage-gated sodium channels
VGKCs: Voltage-gated potassium channels
TLR4: Toll-like receptor 4
TIVA: Total intravenous anesthesia
NSAIDs: Non-steroidal anti-inflammatory drugs
TEA: Thoracic epidural anesthesia
TAP: Transversus abdominis plane
